# Mechanisms of Change in Mindfulness-Based Family Intervention (MYmind) Versus Methylphenidate for Childhood ADHD: A Randomized Controlled Trial

**DOI:** 10.3390/children13030434

**Published:** 2026-03-23

**Authors:** Brett Kosterman Zoller, Susan M. Bögels, Renée Meppelink, Esther I. de Bruin

**Affiliations:** 1 Research Institute of Child Development and Education (RICDE), University of Amsterdam, 1012 WP Amsterdam, The Netherlands; 2 Developmental Psychology, University of Amsterdam, 1012 WP Amsterdam, The Netherlands; 3 Arkin Youth and Family, 1058 AA Amsterdam, The Netherlands; 4 UvA Minds, Academic Treatment Centre, University of Amsterdam, 1071 JW Amsterdam, The Netherlands

**Keywords:** mindfulness, MYmind, methylphenidate, mediation, mechanisms of change, parenting, emotion regulation, self-compassion, self-regulation

## Abstract

**Background/Objectives**: Mindfulness-based interventions show promise for treating childhood ADHD, yet the mechanisms through which they produce effects remain unclear. This study provides the first direct comparison of treatment mechanisms between a mindfulness-based family intervention (MYmind) and methylphenidate. **Methods:** Data were drawn from a preregistered trial combining randomized and preference arms, comparing MYmind (2-month parallel parent–child mindfulness training) with 4-month methylphenidate in children aged 8–18 with ADHD (N = 120 children, 224 parents). Families were assessed at baseline, 2, 4 and 10 months. Multilevel mediation analyses tested whether treatment effects on ADHD symptoms were transmitted through 111 treatment-to-mediator-to-outcome pathways across three mechanism categories: child emotion regulation and coping (all children); adolescent self-regulation and mindfulness (ages 11+); and parent-level mechanisms, including mindful parenting, parental mindfulness, parenting style and self-compassion. **Results:** Direct treatment effects favored methylphenidate for ADHD symptom reduction at 4 months, with mindfulness catching up by 10 months. MYmind produced significantly greater improvements than methylphenidate in adolescent healthy self-regulation, parental self-compassion, mindful parenting and over-reactive parenting. Treatment did not differentially affect the remaining mechanisms. Across model sets, observed emotion regulation, maladaptive coping, parental self-compassion and mindful parenting each predicted ADHD outcomes. Across 111 pathways tested in 18 models, numerous significant individual pathways were consistent with theoretical predictions, yet no complete mediation chains reached statistical significance. **Conclusions:** MYmind engages distinct psychological and family-level processes compared to methylphenidate that are separately associated with ADHD symptom improvement. The absence of significant mediation effects likely reflects power limitations. These findings support mindfulness-based family intervention as a viable alternative to medication and highlight the need for larger-scale mechanism research.

## 1. Introduction

### 1.1. ADHD Prevalence and Family Impact

ADHD affects an estimated 5–7% of children and adolescents worldwide [1,2], with recent U.S. estimates as high as 11.4% [3]. The condition is defined by developmentally inappropriate inattention and/or hyperactivity–impulsivity, causing cross-setting impairment [4], but ADHD also involves significant impairment in academic achievement, peer relationships and family functioning [5,6]. Approximately 77.9% meet criteria for at least one comorbid condition [3], most commonly oppositional defiant disorder, anxiety and depression.

Parenting practices both shape and are shaped by children’s ADHD symptoms, making them a relevant treatment target. Parents of children with ADHD report elevated parenting stress, which is associated with less effective parenting practices [7,8]. Children referred with ADHD are substantially more likely to have a parent with ADHD symptoms (40–50%) [9], and parental ADHD is itself associated with harsher and more lax parenting [10], as well as greater parental over-reactivity [11,12]. This creates a transactional dynamic [13] in which the child’s challenging behaviors elicit more negative parenting responses [14,15,16]. Over time, negative parenting interactions can compromise the child’s developing capacity for self-regulation [15,17]. This cycle may be especially consequential in children already vulnerable to ADHD, for whom regulatory abilities appear particularly dependent on consistent parenting [18]. A meta-analysis of clinical ADHD samples found that negative parenting practices were significantly associated with symptom severity, whereas positive parenting behaviors were not [19]. Still, improvements in both positive and negative parenting mediated treatment effects on child impairment in one trial, independent of changes in inattention [20].

Children with ADHD are also typically more emotionally reactive and labile than their peers, scoring nearly one standard deviation higher on this dimension [21]. Such emotional intensity, combined with noncompliance, can fuel conflictual parent–child interactions. Longitudinal research indicates that coercive parenting and children’s oppositional behavior show reciprocal influences that become entrenched over time [22,23].

### 1.2. Emotion Regulation as a Core Feature of ADHD

Emotion dysregulation is not just a consequence of disrupted parent–child dynamics. It is a substantial feature of ADHD. In a meta-analysis of over 32,000 youth, Graziano and Garcia [21] found large impairments across multiple domains of emotional functioning, with the strongest effects for emotional reactivity and lability (d = 0.95) and emotion regulation (d = 0.80). This pattern persists into adulthood, where emotion dysregulation distinguishes adults with ADHD from healthy controls at g = 1.17 [24] (N = 2535). Prevalence estimates suggest 25–45% of children and 30–70% of adults with ADHD experience clinically significant emotion dysregulation [25].

These difficulties appear to be more than byproducts of core ADHD symptoms. Emotion dysregulation contributes to functional impairment independently of inattention; hyperactivity–impulsivity [26]; and other executive function deficits, including working memory and delay aversion [27]. It also accounts for the association between ADHD symptomatology and adverse outcomes such as symptoms of depression and anxiety, difficulties in romantic and social relationships, and broader functional impairment [28]. Barkley [29] has argued that deficient emotional self-regulation should be considered a core component of ADHD, alongside deficient behavioral and cognitive self-regulation. His view is supported by evidence suggesting that EI and DESR show enough diagnostic specificity to merit consideration as criteria for ADHD [30]. If emotion dysregulation is integral to the disorder rather than secondary to it, then interventions that target emotion regulation directly, rather than only behavioral compliance, may address a more fundamental source of impairment.

### 1.3. The Treatment Landscape: What Works

Two approaches to treating pediatric ADHD have the strongest evidence base: stimulant medication (most commonly methylphenidate) and psychosocial treatments, including behavioral parent training, classroom management, skills-based interventions and, more recently, mindfulness-based interventions. Network meta-analyses have established that stimulants produce robust effects on core ADHD symptoms [31], while behavioral management interventions demonstrate medium-to-large effects on both symptoms and functional impairments [32,33,34].

### 1.4. The Mechanisms Gap

Far less clear is how these treatments produce their effects. Despite decades of ADHD intervention research, formal tests of treatment mechanisms remain scarce. Van der Oord and Daley [35] found “very few” studies testing mediation models for behavioral interventions and “no studies” examining mechanisms of cognitive training. The limited evidence that existed focused almost exclusively on parenting, with reductions in negative or ineffective parenting emerging as the most consistent mediator [36,37]. Little has changed since. DuPaul et al. [38] identified mechanism research as the first priority for advancing psychosocial treatment, and Sibley et al. [39] observed that “almost no studies identify mediators of psychosocial interventions for ADHD—largely due to design limitations” (p. 545), with “no formal tests” of whether youth skill development operates as a mechanism of change. The relative neglect of ADHD is underscored by Taubner et al.’s [40] systematic review of mediators across 106 adolescent psychotherapy RCTs: anxiety (k = 19), depression (k = 20) and substance-use disorders (k = 25) were well-represented, while ADHD was grouped among conditions with only one to three available studies.

Sibley et al.’s [39] data analysis of four RCTs (N = 854) investigated psychosocial treatment mediators for ADHD. Their findings revealed that improvements in organization and time management skills, as well as parent–teen communication, mediated outcomes across treatments, while parent contingency management and disruptive classroom behavior did not. For mindfulness-based interventions (MBIs) specifically, mechanism research remains limited. In the only study we are aware of that examined mediation, in adults with ADHD, it was found that self-compassion, but not mindfulness skills, mediated improvements in positive mental health [41]. No studies to our knowledge have tested mechanisms of MBIs in children or in family-based protocols that target both parent and child.

Indeed, a striking finding across MBI meta-analyses is that these interventions improve ADHD symptoms without necessarily improving measured mindfulness skills. Kim and Jung [42] found that while MBIs significantly improved ADHD symptoms, they did not significantly improve mindfulness skills (SMD = −0.20, ns). Similarly, Lee et al. [43] found that child mindfulness did not significantly improve despite symptom reduction (g = 0.43, ns). Siebelink et al. [44] likewise found no positive effect on children’s self-reported mindfulness skills despite improvements in ADHD symptoms, consistent with earlier findings that dispositional mindfulness and ADHD-related attention represent phenotypically and genetically distinct constructs [45].

This pattern raises a fundamental question: if MBIs improve ADHD symptoms but do not improve mindfulness as measured by self-report scales, what mechanisms account for the observed treatment effects? Several explanations have been proposed. Kosterman Zoller et al. [46] suggested that “perhaps this lack of difference in improvements of mindfulness skills between the groups may be related to the choice of measurement; the Child and Adolescent Mindfulness Measure (CAMM) measures trait mindfulness. To develop mindfulness as an internal trait might be a longer-term process” (pp. 10–11). They also noted that participants’ understanding of mindfulness may shift after training, a response-shift phenomenon. Alternatively, MBIs may work primarily through other pathways, such as improvements in child emotion regulation, reductions in parenting stress or enhanced parent–child relationships in family mindfulness interventions, rather than through changes in mindfulness per se.

Multiple recent reviews have explicitly called for mechanism research to address these questions. Kim and Jung [42] called for research to clarify mechanisms through which mindfulness interventions produce their effects. Lee et al. [43] recommended that researchers “examine the mechanisms of mindfulness in the improvement of ADHD symptoms.” And Siebelink et al. [44] emphasized the importance of further research into the moderators and mediators of MBIs. A better understanding of the mechanisms through which psychosocial treatments for children with ADHD and their parents work can help improve the efficacy of such treatments, inform treatment personalization and clarify which intervention components are essential versus incidental.

### 1.5. Methylphenidate: First-Line Pharmacological Treatment

Among medications for ADHD, including amphetamines and non-stimulants such as atomoxetine, stimulant medication is currently recommended as the first-line pharmacological treatment for children and adolescents with ADHD [47,48]. The medication acts by blocking dopamine and norepinephrine reuptake, thereby increasing catecholamine availability in the prefrontal cortex [49]. Meta-analytic evidence supports the efficacy of methylphenidate for reducing core ADHD symptoms [49], with emerging evidence suggesting benefits for emotional dysregulation [50,51], social functioning [52] and internalizing symptoms [53].

Methylphenidate treatment does however have notable limitations. Long-term effectiveness remains controversial [54], with evidence suggesting that efficacy may diminish over time [55] and that some patients develop tolerance to stimulant medication [56]. An estimated 20–30% of children do not respond adequately to stimulant medication [57]. Common side effects include appetite suppression, sleep difficulties and mood changes [49].

These pharmacological mechanisms are fundamentally different from those targeted by psychosocial interventions, which aim to produce change through skill acquisition, cognitive restructuring or improved family functioning [39]. Medication targets the child’s neurochemistry without directly addressing the family context in which symptoms are expressed and maintained. Whether pharmacological and psychosocial treatments engage complementary or overlapping mechanisms remains largely untested [39,58].

### 1.6. Mindfulness-Based Interventions for ADHD

Mindfulness has been defined as “the awareness that emerges through paying attention on purpose, in the present moment and nonjudgmentally to the unfolding of experience moment by moment” [59] (p. 145). Bishop et al. [60] proposed a two-component model of mindfulness comprising (1) self-regulation of attention to maintain focus on immediate experience, and (2) adopting a particular orientation toward that experience characterized by curiosity, openness and acceptance. Attentional self-regulation is directly relevant to ADHD given that difficulties with sustained and flexible attention are among its defining features [60,61]. Openness and acceptance may additionally support the emotion regulation difficulties described above. Meta-analyses, in fact, support the efficacy of mindfulness-based interventions for reducing ADHD symptoms in both children and adults [42,43], though the mechanisms through which they produce these effects remain poorly understood.

### 1.7. The MYmind Protocol

MYmind is a mindfulness-based intervention developed specifically for children and adolescents with ADHD and related externalizing problems, delivered in parallel to their parents [11]. The protocol consists of eight weekly 90 min sessions for children and a parallel eight-session program for parents, with a booster session at approximately 16 weeks. Child sessions adapt mindfulness practices to address the attention, impulsivity and emotion regulation difficulties characteristic of ADHD. It teaches skills such as focused attention, awareness of impulses before acting on them and non-reactive observation of emotions. The parallel parent program teaches mindful parenting (bringing present-moment, nonjudgmental awareness to parent–child interactions) with the goal of reducing automatic reactive parenting patterns that can maintain coercive cycles [62]. Both children and parents are asked to practice mindfulness daily between sessions. This dual-target structure distinguishes MYmind from medication, which addresses only children’s symptoms. By engaging both parent and child, the intervention has the potential to operate through multiple pathways simultaneously.

### 1.8. Proposed Mechanisms of Change

MYmind’s theoretical model proposes several distinct pathways through which the intervention may improve child outcomes.

#### 1.8.1. Child and Adolescent Self-Regulation

Beyond attention, mindfulness training targets how children regulate emotions. By practicing non-reactive awareness of emotional states, children may learn to notice emotions without immediately acting on them. This is particularly relevant for children with ADHD, for whom emotional impulsivity is a prominent source of impairment [25,61]. Through repeated practice, this may reduce reactive responses such as emotional outbursts, withdrawal or aggression. More broadly, mindfulness training may improve self-regulation as a whole [63]. Adolescents who develop greater dispositional mindfulness may become better able to pause before impulsive responses and respond to events more flexibly rather than reactively [63,64].

#### 1.8.2. Mindful Parenting and Parenting Style

The parallel parent and child component targets change at the family level. Duncan et al. [65] proposed a model of mindful parenting comprising five interrelated dimensions: listening with full attention, nonjudgmental acceptance of self and child, emotional awareness of self and child, self-regulation in the parenting relationship, and compassion for self and child. This last dimension draws on the broader construct of self-compassion, which involves responding to one’s own hardship with kindness rather than harsh self-criticism and holding painful thoughts in mindful awareness rather than over-identifying with them [66]. Bögels et al. [67] further elaborated mechanisms through which mindful parenting may improve parent–child interactions, including reducing parental stress and reactivity, improving parental executive functioning and fostering self-compassion. An open trial of mindful parenting in a clinical sample found improvements in parenting stress, parenting behaviors and child psychopathology, providing preliminary support for these proposed mechanisms [68].

By cultivating mindful awareness and self-compassion, parents may become better able to interrupt automatic reactive parenting patterns. Rather than responding to challenging child behavior through cognitive filters shaped by past frustrations or future worries, parents can respond to the present moment with lower emotional reactivity. If this shift occurs, it should be observable as reductions in overreactive and lax discipline. Parenting behavior thus represents a downstream pathway through which the parent training component may affect child outcomes.

### 1.9. The Present Study

We use data from a randomized controlled trial comparing MYmind and methylphenidate to test whether these treatments produce their effects through different pathways. Using multilevel mediation analysis across multiple timepoints, we examine theoretically derived mediators organized into three model sets.

#### 1.9.1. Model Set 1: Child Emotion Regulation (All Children)

We test whether treatment effects on ADHD symptoms are mediated by improvements in children’s observed mindful emotion regulation and coping strategies (adaptive and maladaptive). We hypothesize that MYmind, but not methylphenidate, will operate through improvements in child emotion regulation.

#### 1.9.2. Model Set 2: Adolescent Self-Regulation (Adolescents 11+)

For older youth capable of reliable self-report, we additionally test whether effects are mediated by improvements in dispositional mindfulness and healthy self-regulation. These two constructs could not be assessed via self-report in younger children and are tested alongside the emotion regulation mediators from Model Set 1. We hypothesize that MYmind will operate through these self-regulatory pathways.

#### 1.9.3. Model Set 3: Parent-Level Mechanisms (Parents)

We test whether treatment effects are mediated by improvements in parents’ own mindfulness, mindful parenting, self-compassion and parenting style (overreactive and lax parenting). We hypothesize that MYmind will operate through these family-level pathways, while methylphenidate will not.

For each model set, we examine mediation at short-term, medium-term and long-term follow-up (see Table 1). Outcomes include parent-reported inattention, hyperactivity/impulsivity and attention problems. In total, we test 111 indirect pathways across three model sets, three outcome variables and three timepoints.

**Table 1:** Overview of mediation models and indirect pathways tested.

Panel A. Models by Model Set and Temporal Period					
Model Set	Temporal Period			Outcomes	Models
All Children (Table 2)	Early (baseline→2 months)			DBDRS Att, DBDRS H-I, CBCL Att	3
	Sustained (baseline→4 months)			DBDRS Att, DBDRS H-I, CBCL Att	3
Adolescents (Table 3)	Early (baseline→2 months)			DBDRS Att, DBDRS H-I, CBCL Att	3
	Sustained (baseline→4 months)			DBDRS Att, DBDRS H-I, CBCL Att	3
Parents (Table 4)	Early (baseline→2 months)			DBDRS Att, DBDRS H-I, CBCL Att	3
	Sustained (baseline→4 months)			DBDRS Att, DBDRS H-I, CBCL Att	3
Total					18
Panel B. Indirect Pathways by Model Set					
Model Set	Temporal	Mediators	Outcomes	Timepoints	Pathways
All Children	Early	3	3	2 (4 months, 10 months)	18
	Sustained	2	3	1 (10 months)	6
	Subtotal				24
Adolescents	Early	5	3	2 (4 months, 10 months)	30
	Sustained	4	3	1 (10 months)	12
	Subtotal				42
Parents	Early	5	3	2 (4 months, 10 months)	30
	Sustained	5	3	1 (10 months)	15
	Subtotal				45
Total					111

Note. Pathways = Mediators × Outcomes × Timepoints. Early models examined mediator changes from baseline to 2 months, predicting outcomes at 4 months and 10 months. Sustained models examined mediator changes from baseline to 4 months, predicting outcomes at 10 months. All Children mediators: Observed Mindful Emotion Regulation, Adaptive Coping, Maladaptive Coping. Adolescent mediators added: CAMM (mindfulness) and HSR (healthy self-regulation). Parent mediators: Over-reactive parenting, lax parenting, FFMQ (mindfulness), IM-P (mindful parenting), Self-Compassion. Outcomes: DBDRS Att = Disruptive Behavior Disorder Rating Scale—Inattention; DBDRS H-I = Disruptive Behavior Disorder Rating Scale—Hyperactivity/Impulsivity; CBCL Att = Child Behavior Checklist—Attention Problems.

**Table 2:** Multilevel mediation results for all children emotion regulation models (Model Set 1).

Outcome	Temporal	Mediator	N	a-Path B (SE)	b-Path 4 Months B (SE)	b-Path 10 Months B (SE)	c′ 4 Months B (SE)	c′ 10 Months B (SE)	Indirect 4 Months	Indirect 10 Months
DBDRS Att	Early	Mindful ER	210/112	−0.013 (0.134)	0.432 (0.318)	0.122 (0.332)	−1.202 (0.265) ***	−0.315 (0.259)	−0.006 (0.057)	−0.002 (0.015)
		Adaptive Coping		−2.432 (1.828)	−0.005 (0.013)	−0.004 (0.013)			0.011 (0.031)	0.010 (0.032)
		Maladaptive Coping		−1.996 (1.414)	0.010 (0.019)	0.033 (0.016) *			−0.019 (0.040)	−0.066 (0.055)
		Total Indirect		---	---	---			−0.014 (0.071)	−0.057 (0.061)
DBDRS H-I	Early	Mindful ER	210/112	−0.003 (0.138)	0.147 (0.261)	−0.225 (0.258)	−1.368 (0.220) ***	−0.597 (0.224) **	0.000 (0.020)	0.001 (0.032)
		Adaptive Coping		−2.499 (1.835)	−0.018 (0.013)	−0.016 (0.011)			0.046 (0.049)	0.040 (0.045)
		Maladaptive Coping		−1.923 (1.412)	0.006 (0.017)	0.015 (0.014)			−0.011 (0.033)	−0.028 (0.033)
		Total Indirect		---	---	---			0.034 (0.058)	0.013 (0.062)
CBCL Att	Early	Mindful ER	210/112	−0.012 (0.133)	0.424 (1.396)	0.434 (1.866)	−4.318 (1.228) ***	−2.153 (1.352)	−0.005 (0.051)	−0.005 (0.053)
		Adaptive Coping		−2.478 (1.823)	−0.090 (0.066)	−0.076 (0.066)			0.223 (0.222)	0.188 (0.198)
		Maladaptive Coping		−1.982 (1.419)	−0.032 (0.090)	0.088 (0.085)			0.064 (0.194)	−0.175 (0.207)
		Total Indirect		---	---	---			0.282 (0.285)	0.008 (0.248)
DBDRS Att	Sustained	Adaptive Coping	205/109	3.574 (2.087) ^†^	---	−0.007 (0.012)	---	−0.306 (0.239)	---	−0.024 (0.046)
		Maladaptive Coping		−0.509 (1.560)	---	0.043 (0.017) *			---	−0.022 (0.065)
		Total Indirect		---	---	---			---	−0.046 (0.082)
DBDRS H-I	Sustained	Adaptive Coping	205/109	3.724 (2.083) ^†^	---	0.010 (0.011)	---	−0.596 (0.217) **	---	0.039 (0.047)
		Maladaptive Coping		−0.683 (1.566)	---	0.000 (0.016)			---	0.000 (0.011)
		Total Indirect		---	---	---			---	0.039 (0.052)
CBCL Att	Sustained	Adaptive Coping	205/109	3.504 (2.080) ^†^	---	−0.090 (0.069)	---	−2.047 (1.323)	---	−0.314 (0.319)
		Maladaptive Coping		−0.550 (1.565)	---	0.202 (0.118) ^†^			---	−0.111 (0.303)
		Total Indirect		---	---	---			---	−0.425 (0.428)

Note. N = observations/families for multilevel models. DBDRS = Disruptive Behavior Disorders Rating Scale; H-I = Hyperactivity–Impulsivity; CBCL = Child Behavior Checklist; Att = Attention Problems; Mindful ER = Observed Mindful Emotion Regulation). Treatment coded as 1 = Mindfulness, 2 = Medication. Early models examine baseline→2 months mediator changes predicting outcomes at 4 months and 10 months. Sustained models examine baseline→4 months mediator changes predicting 10 months outcomes only. Dashes indicate pathways not tested in sustained designs. All coefficients are unstandardized. Column definitions: a-path = effect of treatment on mediator (positive = mindfulness shows greater improvement); b-path = effect of mediator change on outcome, controlling for treatment; c′ = direct effect of treatment on outcome after controlling for all mediators (residual treatment effect not explained by mechanisms); Indirect = a × b, the effect of treatment on outcome transmitted through each mediator. ^†^ *p* < 0.10. * *p* < 0.05. ** *p* < 0.01. *** *p* < 0.001. Key Findings: Zero of 24 specific indirect effects (9 at 4 months and 15 at 10 months) reached statistical significance. Treatment significantly predicted attention outcomes at 4 months across all three measures: DBDRS Attention (B = −1.202, *p* < 0.001), DBDRS Hyperactivity (B = −1.368, *p* < 0.001) and CBCL Attention (B = −4.318, *p* < 0.001). Treatment also predicted DBDRS Hyperactivity at 10 months (B = −0.597, *p* = 0.008). For sustained effects, maladaptive coping predicted DBDRS Attention at 10 months (B = 0.043, *p* = 0.012). Sustained adaptive coping changes showed marginal treatment effects across all outcomes (Bs = 3.50–3.72, ps = 0.074–0.092).

**Table 3:** Multilevel mediation results for subsample adolescent self-regulation models (Model Set 2).

Outcome	Temporal	Mediator	N	a-Path B (SE)	b-Path 4 Months B (SE)	b-Path 10 Months B (SE)	c′ 4 Months B (SE)	c′ 10 Months B (SE)	Indirect 4 Months	Indirect 10 Months
DBDRS Att	Early	Mindful ER	88/48	−0.044 (0.184)	0.760 (0.356) *	0.646 (0.358) †	−0.717 (0.486)	−0.279 (0.451)	−0.034 (0.136)	−0.029 (0.112)
		Adaptive Coping		−2.554 (2.854)	−0.032 (0.027)	−0.004 (0.029)			0.082 (0.106)	0.009 (0.073)
		Maladaptive Coping		−2.753 (1.741)	−0.005 (0.037)	−0.002 (0.052)			0.015 (0.101)	0.004 (0.144)
		CAMM		−0.023 (0.164)	0.143 (0.479)	−0.451 (0.586)			−0.003 (0.025)	0.010 (0.078)
		HSR		0.439 (0.197) *	0.148 (0.360)	0.341 (0.355)			0.065 (0.158)	0.150 (0.172)
		Total Indirect		—	—	—			0.124 (0.262)	0.145 (0.273)
DBDRS H-I	Early	Mindful ER	88/48	−0.044 (0.189)	0.234 (0.331)	0.073 (0.420)	−1.212 (0.345) ***	−0.706 (0.444)	−0.010 (0.045)	−0.003 (0.017)
		Adaptive Coping		−2.704 (2.877)	−0.045 (0.024) †	−0.028 (0.024)			0.121 (0.130)	0.076 (0.100)
		Maladaptive Coping		−2.898 (1.732) †	−0.006 (0.034)	−0.023 (0.045)			0.016 (0.099)	0.068 (0.131)
		CAMM		−0.027 (0.172)	0.365 (0.418)	0.077 (0.525)			−0.010 (0.060)	−0.002 (0.015)
		HSR		0.459 (0.203) *	0.047 (0.268)	0.251 (0.310)			0.022 (0.123)	0.115 (0.160)
		Total Indirect		—	—	—			0.139 (0.181)	0.254 (0.206)
CBCL Att	Early	Mindful ER	88/48	−0.025 (0.192)	1.132 (0.912)	2.165 (2.026)	−3.330 (1.441) *	−4.638 (1.411) **	−0.028 (0.214)	−0.054 (0.410)
		Adaptive Coping		−2.188 (2.885)	−0.110 (0.101)	−0.127 (0.103)			0.241 (0.325)	0.279 (0.345)
		Maladaptive Coping		−2.539 (1.745)	−0.247 (0.151)	−0.330 (0.203)			0.628 (0.512)	0.839 (0.706)
		CAMM		−0.020 (0.163)	−1.149 (1.906)	−2.905 (3.006)			0.022 (0.200)	0.057 (0.490)
		HSR		0.420 (0.197) *	1.361 (1.095)	3.739 (1.828) *			0.572 (0.511)	1.570 (1.123)
		Total Indirect		—	—	—			1.434 (0.822) †	2.690 (1.742)
DBDRS Att	Sustained	Adaptive Coping	87/48	2.205 (3.412)	—	−0.020 (0.018)	—	0.079 (0.360)	—	−0.044 (0.072)
		Maladaptive Coping		−1.732 (2.761)	—	0.045 (0.024) †			—	−0.078 (0.110)
		CAMM		−0.137 (0.184)	—	−0.496 (0.401)			—	0.068 (0.106)
		HSR		0.146 (0.184)	—	−0.381 (0.300)			—	−0.056 (0.071)
		Total Indirect		—	—	—			—	−0.109 (0.203)
DBDRS H-I	Sustained	Adaptive Coping	87/48	2.457 (3.379)	—	0.013 (0.017)	—	−0.630 (0.421)	—	0.032 (0.064)
		Maladaptive Coping		−2.278 (2.708)	—	−0.038 (0.021) †			—	0.086 (0.116)
		CAMM		−0.116 (0.187)	—	−0.112 (0.434)			—	0.013 (0.059)
		HSR		0.158 (0.184)	—	0.237 (0.351)			—	0.037 (0.071)
		Total Indirect		—	—	—			—	0.169 (0.189)
CBCL Att	Sustained	Adaptive Coping	87/48	2.440 (3.356)	—	−0.046 (0.081)	—	−2.690 (1.421) †	—	−0.113 (0.252)
		Maladaptive Coping		−2.152 (2.676)	—	−0.036 (0.135)			—	0.078 (0.339)
		CAMM		−0.091 (0.184)	—	−4.216 (2.229) †			—	0.385 (0.841)
		HSR		0.144 (0.182)	—	2.660 (1.171) *			—	0.384 (0.544)
		Total Indirect		—	—	—			—	0.733 (1.101)

Note. N = observations/families for multilevel models. DBDRS = Disruptive Behavior Disorders Rating Scale; H-I = Hyperactivity–Impulsivity; CBCL = Child Behavior Checklist; Att = Attention Problems; Mindful ER = Observed Mindful Emotion Regulation (AddER); CAMM = Child and Adolescent Mindfulness Measure; HSR = Healthy Self-Regulation. Treatment coded as 1 = Mindfulness, 2 = Medication. Early models examine baseline→2 months mediator changes predicting outcomes at 4 months and 10 months. Sustained models examine baseline→4 months mediator changes predicting 10 months outcomes only. Dashes indicate pathways not tested in sustained designs. All coefficients are unstandardized. Column definitions: a-path = effect of treatment on mediator (positive = mindfulness shows greater improvement); b-path = effect of mediator change on outcome, controlling for treatment; c′ = direct effect of treatment on outcome after controlling for all mediators (residual treatment effect not explained by mechanisms); Indirect = a × b, the effect of treatment on outcome transmitted through each mediator. † *p* < 0.10. * *p* < 0.05. ** *p* < 0.01. *** *p* < 0.001. Key Findings: Zero of 42 specific indirect effects (15 at 4 months and 27 at 10 months) reached statistical significance. Treatment significantly predicted increases in Healthy Self-Regulation (HSR) in early models (Bs = 0.420–0.459, ps = 0.024–0.033). Early HSR increases predicted more CBCL attention problems at 10 months (B = 3.739, *p* = 0.041). Sustained HSR increases also predicted more CBCL attention problems at 10 months (B = 2.660, *p* = 0.023). Early Mindful ER increases predicted better DBDRS attention at 4 months (B = 0.760, *p* = 0.033). Sustained CAMM changes showed a marginal effect on CBCL attention (B = −4.216, *p* = 0.059).

**Table 4:** Parent-level mediation models: Treatment effects on child ADHD symptoms through parent mechanisms (Model Set 3).

Outcome	Temporal	Mediator	N	a-Path B (SE)	b-Path 4 Months B (SE)	b-Path 10 Months B (SE)	c′ 4 Months B (SE)	c′ 10 Months B (SE)	Indirect 4 Months	Indirect 10 Months
DBDRS H-I	Early	Over-Reactive	147/112	0.176 (0.096) ^†^	0.027 (0.322)	0.321 (0.273)	−1.581 (0.270) ***	−0.623 (0.250) *	0.005 (0.057)	0.056 (0.062)
		Lax		−0.050 (0.093)	−0.186 (0.287)	−0.444 (0.234) ^†^			0.009 (0.024)	0.022 (0.045)
		FFMQ		−0.013 (0.049)	0.685 (0.606)	0.231 (0.617)			−0.009 (0.034)	−0.003 (0.013)
		IM-P		−0.065 (0.038) ^†^	−1.611 (0.691) *	0.011 (0.729)			0.105 (0.074)	−0.001 (0.047)
		Self-Compassion		−0.376 (0.123) **	−0.083 (0.235)	−0.073 (0.238)			0.031 (0.091)	0.027 (0.092)
DBDRS Att	Early	Over-Reactive	147/112	0.162 (0.096) ^†^	−0.345 (0.369)	0.058 (0.293)	−1.451 (0.319) ***	−0.513 (0.296) ^†^	−0.056 (0.063)	0.009 (0.049)
		Lax		−0.050 (0.092)	−0.051 (0.311)	0.170 (0.289)			0.003 (0.017)	−0.008 (0.018)
		FFMQ		−0.013 (0.049)	0.071 (0.727)	0.065 (0.690)			−0.001 (0.009)	−0.001 (0.009)
		IM-P		−0.063 (0.038)	−0.326 (1.087)	−0.128 (0.792)			0.020 (0.072)	0.008 (0.050)
		Self-Compassion		−0.378 (0.122) **	−0.358 (0.314)	−0.195 (0.280)			0.136 (0.130)	0.074 (0.110)
CBCL Att	Early	Over-Reactive	148/113	0.167 (0.096) ^†^	−2.108 (1.568)	−0.680 (1.532)	−5.033 (1.317) ***	−2.694 (1.320) *	−0.351 (0.316)	−0.113 (0.259)
		Lax		−0.049 (0.093)	−1.372 (1.351)	−1.026 (1.386)			0.067 (0.156)	0.050 (0.126)
		FFMQ		−0.012 (0.049)	2.166 (2.704)	0.421 (2.862)			−0.027 (0.105)	−0.005 (0.039)
		IM-P		−0.065 (0.038) ^†^	−8.181 (3.770) *	−5.858 (3.372) ^†^			0.531 (0.412)	0.380 (0.308)
		Self-Compassion		−0.374 (0.122) **	0.150 (1.067)	0.168 (1.017)			−0.056 (0.399)	−0.063 (0.384)
DBDRS H-I	Sustained	Over-Reactive	139/107	0.228 (0.084) **	—	0.306 (0.292)	—	−0.468 (0.253) ^†^	—	0.070 (0.070)
		Lax		−0.059 (0.091)	—	0.035 (0.243)			—	−0.002 (0.015)
		FFMQ		−0.056 (0.048)	—	0.163 (0.377)			—	−0.009 (0.022)
		IM-P		−0.113 (0.045) *	—	0.987 (0.613)			—	−0.112 (0.082)
		Self-Compassion		−0.197 (0.124)	—	−0.111 (0.216)			—	0.022 (0.044)
DBDRS Att	Sustained	Over-Reactive	139/107	0.229 (0.084) **	—	0.003 (0.350)	—	−0.487 (0.276) ^†^	—	0.001 (0.080)
		Lax		−0.059 (0.091)	—	0.244 (0.297)			—	−0.014 (0.028)
		FFMQ		−0.056 (0.048)	—	−0.441 (0.461)			—	0.025 (0.033)
		IM-P		−0.113 (0.045) *	—	0.625 (0.765)			—	−0.070 (0.095)
		Self-Compassion		−0.194 (0.124)	—	−0.629 (0.244) *			—	0.122 (0.092)
CBCL Att	Sustained	Over-Reactive	139/107	0.229 (0.085) **	—	−0.356 (1.286)	—	−3.116 (1.489) *	—	−0.082 (0.291)
		Lax		−0.059 (0.091)	—	−0.484 (1.282)			—	0.028 (0.085)
		FFMQ		−0.056 (0.048)	—	1.374 (2.374)			—	−0.076 (0.148)
		IM-P		−0.114 (0.045) *	—	−4.651 (2.632) ^†^			—	0.530 (0.337)
		Self-Compassion		−0.198 (0.123)	—	−0.536 (1.270)			—	0.106 (0.237)

Note. N = observations/families for multilevel models. DBDRS = Disruptive Behavior Disorders Rating Scale; H-I = Hyperactivity–Impulsivity; CBCL = Child Behavior Checklist; Att = Attention Problems; FFMQ = Five-Facet Mindfulness Questionnaire; IM-P = Interpersonal Mindfulness in Parenting. Treatment coded as 1 = Mindfulness, 2 = Medication. Early models examine baseline→2 months mediator changes predicting outcomes at 4 months and 10 months. Sustained models examine baseline→4 months mediator changes predicting 10 months outcomes only. Dashes indicate pathways not tested in sustained designs. All coefficients are unstandardized. Column definitions: a-path = effect of treatment on mediator (positive = mindfulness shows greater improvement); b-path = effect of mediator change on outcome, controlling for treatment; c′ = direct effect of treatment on outcome after controlling for all mediators (residual treatment effect not explained by mechanisms); Indirect = a × b, the effect of treatment on outcome transmitted through each mediator. ^†^ *p* < 0.10. * *p* < 0.05. ** *p* < 0.01. *** *p* < 0.001. Key Findings: Zero of 45 specific indirect effects (15 at 4 months and 30 at 10 months) reached statistical significance. Treatment significantly predicted early self-compassion increases (Bs = −0.374 to −0.378, ps < 0.01) and sustained over-reactive parenting reductions (Bs = 0.228–0.229, ps < 0.01) and IM-P increases (Bs = −0.113 to −0.114, ps < 0.05). Significant b-paths emerged for IM-P predicting DBDRS Hyperactivity at 4 months (B = −1.611, *p* < 0.05) and CBCL Attention at 4 months (B = −8.181, *p* < 0.05) in early models and for self-compassion predicting DBDRS Attention at 10 months (B = −0.629, *p* < 0.05) in sustained models. Direct treatment effects favoring medication remained significant at 4 months across all early models: DBDRS Hyperactivity (B = −1.581, *p* < 0.001), DBDRS Attention (B = −1.451, *p* < 0.001) and CBCL Attention (B = −5.033, *p* < 0.001), with effects persisting at 10 months for DBDRS Hyperactivity (B = −0.623, *p* < 0.05) and CBCL Attention (B = −2.694, *p* < 0.05). In sustained models, the direct effect remained significant only for CBCL Attention (B = −3.116, *p* < 0.05).

## 2. Materials and Methods

### 2.1. Participants

Families were recruited through referrals to two outpatient mental health centers via general practitioners and mental health professionals, as well as through the study website and local media. Children were eligible if they were aged 8–18 years, met DSM-based criteria for ADHD as assessed by the Structured Clinical Interview for DSM-5 -Junior (SCID-Junior) [69] and had an IQ above 80. At least one parent was required to participate. Exclusion criteria included inadequate Dutch language proficiency; a history of psychosis, schizophrenia or untreated posttraumatic stress disorder; severe conduct problems that precluded valid assessment; current participation in a psychosocial intervention and methylphenidate use; or mindfulness training participation within the previous 12 months.

To maximize statistical power, we combined data from the randomized controlled trial (RCT; n = 91 children) and a parallel preference trial (PT; n = 29 children). Families who refused randomization because of strong treatment preferences were permitted to select their condition and were assigned to the PT to address ethical concerns about withholding preferred treatment. Ultimately, treatment results were similar between both RCT and PT. For the present analyses, participants were categorized by treatment actually received at short-term follow-up rather than by original assignment. This was done to increase the number of participants and ultimately statistical power. The combined sample included 120 children (74 boys, 46 girls; M age = 11.4 years, SD = 2.6) and 224 parents. Sample sizes varied across model sets based on specific inclusion criteria: Model Set 1 (all children) included 112 children and 210 parent observations; Model Set 2 (adolescents aged 11+) included 48 adolescents and 87 parent observations; and Model Set 3 (parents who completed short-term assessment and, for those in the mindfulness condition, attended at least one training session) included 147 parents, representing 112 families.

### 2.2. Procedure

**Figure 1:**
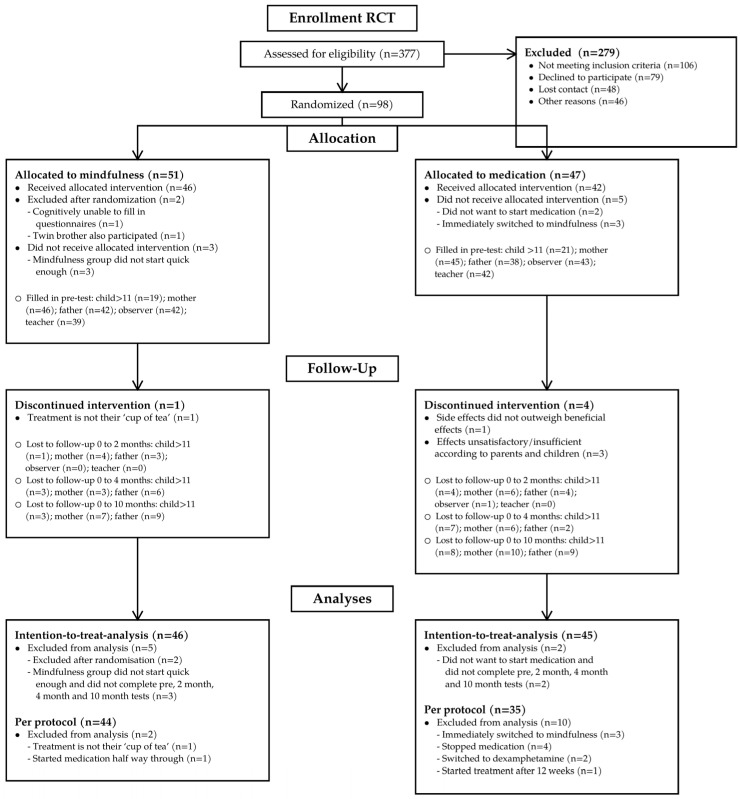
CONSORT flowchart randomized controlled trial.

This preregistered study (https://onderzoekmetmensen.nl/nl/trial/22179, last accessed 20 March 2026) was approved by the Medical Ethics Committee of Amsterdam Medical Center. Parents provided written informed consent and children aged 12 years and older provided assent. For the RCT, families were allocated to treatment condition using computer-generated stratified randomization. The study was conducted and reported in accordance with CONSORT 2010 [70]. Please see Figure 1 below (adapted from Meppelink et al. [71]). Reporting completeness was additionally assessed with the TREND checklist [72]. The completed TREND checklist is provided as Table S1 in the Supplementary Materials.

Data were collected at four timepoints: baseline, short-term follow-up at 2 months, medium-term follow-up at 4 months and long-term follow-up at 10 months. Questionnaires were completed online at home at all timepoints. Families adhered to their assigned or chosen treatment for the first 4 months, after which they were free to pursue alternative approaches, including the treatment to which they were not originally assigned or other psychosocial interventions. Because families could modify their treatment after 4 months, the study functioned as a comparative efficacy trial only through 4 months. We therefore interpret long-term findings at 10 months with this caveat in mind.

### 2.3. Interventions

#### 2.3.1. Mindfulness-Based Intervention

The MYmind program [73] consisted of 8 weekly 90 min group sessions for children, with a parallel mindful parenting training for parents. Daily home meditation practice was assigned throughout the 8-week training period. Child groups included approximately 6 children, while adolescent groups included 8–10 participants. The adolescent curriculum was adapted to include developmentally appropriate content such as school performance, autonomy, parental conflict and substance use, with slightly longer meditation exercises. Both parents were invited to participate in the parent training; across the combined sample, 20 parent couples, 33 mothers and 9 fathers participated. During the 2 months following the group sessions, families were encouraged to maintain their practice according to an individualized meditation plan developed during the final session. A 90 min booster session was provided 2 months after training completion.

Treatment integrity was assessed using the MYmind Treatment Adherence and Competence Scale (MYmind-TACS) [74]. Sixty-six percent of child sessions and 70% of parent sessions were videotaped for coding. Interrater reliability was established on a subset of seven randomly selected sessions per condition; absolute agreement was 93.2% (child) and 94.7% (parent) for adherence and 73.5% (child) and 86.6% (parent) for competence. Intraclass correlations were excellent (adherence: 0.83 and 0.86; competence: 0.80 and 0.88, for child and parent sessions respectively). Across 50 additional randomly selected sessions per condition, trainers adhered to 85.8% of the child protocol (9.8% partial adherence, 4.4% not delivered) and 91.6% of the parent protocol (3.8% partial, 4.7% not delivered). Deviations were primarily due to time constraints or responsiveness to group needs. Mean trainer competence ratings (5-point scale) were 4.29 (SD = 0.60) for child sessions and 4.60 (SD = 0.27) for parent sessions.

#### 2.3.2. Medication

Short-acting methylphenidate was prescribed and monitored by a psychiatrist following Dutch Multidisciplinary Guidelines for ADHD [75]. Children began with three daily doses of 2.5 or 5 mg, administered 7 days per week. The psychiatrist contacted families weekly by telephone until optimal titration was achieved, with in-person consultations with the child and parent(s) occurring every 4 weeks. Dosage or medication type was adjusted if methylphenidate proved ineffective or if side effects outweighed benefits. In some cases, medication was discontinued.

### 2.4. Measures

#### 2.4.1. Primary Outcomes: Child ADHD Behaviors

##### Disruptive Behavior Disorder Rating Scale—Inattention (DBDRS-I)

Inattention symptoms were assessed using the Inattention subscale of the Dutch version of the Disruptive Behavior Disorders Rating Scale (DBDRS) [76,77]. The DBDRS is a parent-report measure that assesses ADHD symptoms based on DSM-IV criteria. The Inattention subscale consists of 9 items rated on a 4-point scale (0 = not at all to 3 = very much). Example items include “Fails to give close attention to details or makes careless mistakes in schoolwork” and “Has difficulty sustaining attention in tasks or play activities.” Internal reliabilities at pretest, 2 months, 4 months and 10 months were α = 0.82, 0.88, 0.89 and 0.87.

##### Disruptive Behavior Disorder Rating Scale—Hyperactivity/Impulsivity (DBDRS-H/I)

Hyperactivity and impulsivity symptoms were assessed using the Hyperactivity/Impulsivity subscale of the DBDRS [76,77]. This subscale comprises 9 items rated on the same 4-point scale. Example items include “Fidgets with hands or feet or squirms in seat” and “Has difficulty awaiting turn.” Internal reliabilities at pretest, 2 months, 4 months and 10 months were α = 0.84, 0.87, 0.88 and 0.90.

##### Child Behavior Checklist—Attention Problems (CBCL-A)

Attention difficulties were also assessed using the Attention Problems subscale from the Child Behavior Checklist (CBCL) [78], part of the Achenbach System of Empirically Based Assessment (ASEBA). The CBCL is a 113-item parent-report measure of child behavioral and emotional problems rated on a 3-point scale (0 = not true to 2 = very true or often true). The Attention Problems subscale comprises 11 items. Example items include “Can’t concentrate, can’t pay attention for long” and “Daydreams or gets lost in his/her thoughts.” Internal reliabilities at pretest, 2 months, 4 months and 10 months were α = 0.66, 0.77, 0.77 and 0.77.

#### 2.4.2. Model Set 1 Mediators: Child Emotion Regulation and Coping (All Children)

##### Observed Mindful Emotion Regulation

Observed mindful emotion regulation during parent–child interactions was assessed using an adapted version of the Emotion Discussion Task (EDT) [79,80]. Parents were instructed to engage in three 5 min conversations with their child about specific emotions (fear, anger and sadness; [79] originally assessed anxious, angry and happy). The goal was to measure the degree to which the participating parent and child used mindful communication when discussing emotions. For this study, the task was administered at pretest and 2-month follow-up, with interactions between one parent and child being videotaped. Following consultation with the measure’s developer, Cynthia Suveg, videos were analyzed using the Observed Mindful Emotion Regulation scoring form [80], focused on mindfulness aspects in emotion communication between parent and child. Scores ranged from 0 = no characteristics of mindfulness (acceptance, curious attitude, calm, attention, awareness, support and compassion) to 4 = many characteristics of mindfulness. A high score across the three emotions indicated high observed mindful emotion regulation. For the present study, only observed child mindful emotion regulation scores were used. Internal reliabilities at pretest and 2 months were α = 0.92 and 0.94.

##### Adaptive Coping

Children’s adaptive coping strategies were assessed using the adaptive subscales from the Assessment of Emotion Regulation in Children and Adolescents (FEEL-KJ) [81], adapted and validated in Dutch [82]. The FEEL-KJ assesses children’s emotion regulation strategies across three negative emotions (anxiety, anger and sadness) on a 5-point scale (1 = almost never to 5 = almost always). The adaptive coping composite includes seven adaptive strategies: Problem-Oriented Action, Distraction, Mood Elevation, Acceptance, Forgetting, Cognitive Problem-Solving and Revaluation. The measure comprises 42 items: 7 strategies with 6 items each and 2 items per strategy for each of the three emotions (anxiety, anger and sadness). Example items include “When I feel [emotion], I do something to make the situation better”; “When I feel [emotion], I do something fun”; and “When I feel [emotion], I tell myself it’s not that important.” For the present study, the total adaptive coping score (sum across all 42 items) was used and norm-scored based on children’s age and gender. Internal reliabilities at pretest, 2 months, 4 months and 10 months were α = 0.95, 0.97, 0.96 and 0.96.

##### Maladaptive Coping

Children’s maladaptive coping strategies were assessed using the maladaptive subscales from the FEEL-KJ [81,82]. The maladaptive coping composite includes five maladaptive strategies: Giving Up, Aggressive Actions, Withdrawal, Self-Devaluation and Rumination. The measure comprises 30 items: 5 strategies with 6 items each and 2 items per strategy for each of the three emotions. Items are rated on the same 5-point scale as for Adaptive Coping. Example items include “When I feel [emotion], I think I can’t do anything right”; “When I feel [emotion], I just want to be alone”; and “When I feel [emotion], I keep thinking about how unfair it is.” For the present study, the total maladaptive coping score (sum across all 30 items) was used and norm-scored based on children’s age and gender. Internal reliabilities at pretest, 2 months, 4 months and 10 months were α = 0.81, 0.85, 0.85 and 0.89.

#### 2.4.3. Model Set 2 Mediators: Adolescent Self-Regulation (Adolescents Only)

##### Mindfulness (Adolescent)

General mindfulness in adolescents was assessed using the 10-item short form of the Child and Adolescent Mindfulness Measure (CAMM-10) [83,84]. The CAMM is a self-report measure rated on a 5-point scale (0 = never true to 4 = always true). Items assess present-moment awareness and nonjudgmental acceptance. Example items include “I get upset with myself for having feelings that don’t make sense”; “At school, I walk from class to class without noticing what I’m doing” (reverse scored); and “I push away thoughts that I don’t like” (reverse scored). All items are reverse-scored, such that higher scores indicate greater mindfulness. Internal reliabilities at pretest, 2 months, 4 months and 10 months were α = 0.75, 0.79, 0.73 and 0.83.

##### Healthy Self-Regulation (Adolescent)

Healthy Self-Regulation in adolescents was assessed using the Dutch version of the Healthy Self-Regulation scale (HSR-NL) [85,86]. The HSR is a 12-item self-report measure rated on a 6-point scale (1 = almost never to 6 = almost always). The scale assesses adaptive self-regulation, including self-acceptance, attention control, patience and emotional regulation. Example items include “I accept myself, even when I still have things to learn”; “When I realize in the middle of a task that I forgot what I was doing, I can bring my attention back”; “When I’m insulted, I need to take revenge” (reverse scored); and “Others can describe me as patient with myself.” Internal reliabilities at pretest, 2 months, 4 months and 10 months were α = 0.81, 0.79, 0.82 and 0.79.

#### 2.4.4. Model Set 3 Mediators: Parent-Level Mechanisms (Parents Only)

##### Over-Reactive Parenting

Over-reactive parenting was assessed using the Over-Reactivity subscale from the Dutch version of the Parenting Scale (PS) [87,88]. The PS is a 30-item self-report measure assessing dysfunctional discipline practices. The Over-Reactivity subscale comprises 10 items rated on a 7-point scale, with each item presenting two opposing parenting responses. An example item is as follows: “When I’m upset or under stress…” 1 = “I am no more picky than usual” vs. 7 = “I am picky and on my child’s back.” Higher scores indicate more over-reactive parenting. Internal reliabilities at pretest, 2 months, 4 months and 10 months were α = 0.77, 0.77, 0.80 and 0.73.

##### Lax Parenting

Lax parenting was assessed using the Laxness subscale from the Parenting Scale (PS) [87,88]. The Laxness subscale comprises 11 items rated on the same 7-point scale format. Higher scores indicate more permissive or inconsistent parenting. An example item is as follows: “When my child misbehaves…” 1 = “I do something right away” vs. 7 = “I do something about it later.” Internal reliabilities at pretest, 2 months, 4 months and 10 months were α = 0.79, 0.80, 0.81 and 0.81.

##### Mindfulness (Parent)

General mindfulness in parents was assessed using the short form of the Five-Facet Mindfulness Questionnaire (FFMQ-SF) [89], a 24-item self-report measure rated on a 5-point scale (1 = never or very rarely true to 5 = very often or always true). The FFMQ assesses five domains of mindfulness: Observing, Describing, Acting with Awareness, Nonjudging of Inner Experience and Non-Reactivity to Inner Experience. Example items include “I rush through activities without being really attentive to them” (reverse scored); “Usually when I have distressing thoughts or images, I step back and am aware of the thought or image without getting taken over by it”; and “I watch my feelings without getting lost in them.” For the present study, only the total mindfulness score was used. Internal reliabilities at pretest, 2 months, 4 months and 10 months were α = 0.77, 0.83, 0.85 and 0.85.

##### Mindful Parenting

Mindful parenting was assessed using the Dutch version of the Interpersonal Mindfulness in Parenting Scale (IM-P) [68], a 29-item self-report measure rated on a 5-point scale (1 = never true to 5 = always true) and based on the five-dimension model of mindful parenting [65]. The IM-P comprises six subscales: Listening with Full Attention, Compassion for Child, Nonjudgmental Acceptance of Parental Functioning, Emotional Non-reactivity in Parenting, Emotional Awareness of Child and Emotional Awareness of Self. Example items include “I am listening to my child with full attention”; “I am usually unaware of my child’s feelings” (reverse scored); and “When I’m upset with my child, I notice how I am feeling before I take action.” For the present study, only the total mindful parenting score was used. Internal reliabilities at pretest, 2 months, 4 months and 10 months were α = 0.87, 0.89, 0.89 and 0.86.

##### Self-Compassion (Parent)

Parental self-compassion was assessed using the short form of the Self-Compassion Scale (SCS-SF) [90], a 12-item self-report measure rated on a 5-point scale (1 = almost never to 5 = almost always). The SCS-SF comprises six subscales: Self-Kindness, Self-Judgment, Common Humanity, Isolation, Mindfulness and Over-identification. Example items include “When I’m going through a very hard time, I give myself the caring and tenderness I need”; “When I feel inadequate in some way, I try to remind myself that feelings of inadequacy are shared by most people”; and “I’m disapproving and judgmental about my own flaws and inadequacies” (reverse scored). For the present study, only the total self-compassion score was used. Internal reliabilities at pretest, 2 months, 4 months and 10 months were α = 0.85, 0.87, 0.90 and 0.89.

### 2.5. Data Analysis—One-Paragraph Version

Multilevel mediation analyses were conducted in Mplus 8.11 (Muthén & Muthén, Los Angeles, CA, USA) [91] using the product-of-coefficients approach with robust maximum likelihood estimation (MLR). All mediators and outcomes were operationalized as raw change scores, with mediators capturing early (baseline–2 months) or sustained (baseline–4 months) change and outcomes reflecting medium-term (baseline–4 months) or long-term (baseline–10 months) change. Multiple mediators were entered simultaneously so that each specific indirect effect represents the unique contribution of a given mechanism; significance was evaluated using 95% Wald confidence intervals. For Model Sets 1 and 2, parent reports were nested within families using a two-level specification (TYPE = TWOLEVEL RANDOM), while Model Set 3 used cluster-robust standard errors (TYPE = COMPLEX) to account for non-independence of parents within families. Trial type was controlled in all models, and missing data were handled through full information maximum likelihood. In total, 18 models were estimated, yielding 111 specific indirect effects.

Each model has four estimates. The a-path tests between-group differences on each mediator. The b-path tests the link between mediator change and child ADHD outcomes, controlling for treatment condition. Multiplying the coefficients of the two paths (a × b) gives the indirect effect, which captures how much of the treatment difference is explained by a given mediator. For an indirect effect to reach significance, both paths must be strong enough within the model. The c’-path (direct effect) is the between-group difference on child ADHD outcomes that remains after accounting for all mediators. If the c’-path is no longer significant at a later timepoint, the two groups may have converged or treatments may have lost effectiveness. Since each model set includes different mediators and draws on slightly different subsamples, c’-path values may vary.

## 3. Results

We explored whether treatment effects on child ADHD symptoms were mediated by changes in child emotion regulation, adolescent self-regulation and parent-level mechanisms across three model sets. Data from four timepoints (baseline, 2 months, 4 months and 10 months) were analyzed to test 111 specific indirect pathways across 18 models. As described in Section 2.5, each of the 18 models estimates the effect of treatment on each mediator (a-path), the association between mediator change and child outcomes (b-path) and the residual between-group difference after accounting for mediators (direct effect c’-path). The indirect effect is calculated by multiplying the a-path and b-path coefficients (a × b) and represents how much of the treatment effect is explained by a given mediator. All three model sets include different mediators, so the c’-path values are not identical across sets. Each model set contains an early and a sustained model for each of the three child ADHD outcomes, resulting in six models per set. Sample sizes varied across model sets: all children (n = 112), adolescents (n = 48) and parents (n = 147). While significant treatment effects on several mediators and multiple significant associations between mediators and outcomes were found, no indirect effects reached statistical significance. For complete findings of all path coefficients, confidence intervals and model fit statistics, see Table 2, Table 3 and Table 4.

### 3.1. Model Set Outcomes

#### 3.1.1. Model Set 1: Child Emotion Regulation and Coping

We analyzed whether early changes (baseline to 2 months) and sustained changes (baseline to 4 months) in children’s emotion regulation strategies mediated treatment effects on ADHD symptoms. The three mediators examined were observed mindful emotion regulation during video recorded parent–child discussions on fear, anger and sadness; children’s self-reported adaptive coping strategies; and children’s self-reported maladaptive coping strategies.

In the early change models which examine from baseline to 2 months, medication produced significantly greater reductions than MYmind in parent-reported child ADHD symptoms at the 4-month follow-up. Between-group differences (c′) at 4 months were significant for DBDRS inattention (B = −1.202, *p* < 0.001), DBDRS hyperactivity/impulsivity (B = −1.368, *p* < 0.001) and CBCL attention problems (B = −4.318, *p* < 0.001), all favoring medication. At the 10-month follow-up, between-group differences remained significant in favor of medication only for DBDRS hyperactivity/impulsivity (B = −0.597, *p* < 0.01), while those on DBDRS inattention (B = −0.315) and CBCL attention problems (B = −2.153) were no longer significant (see Table 2).

Treatment condition did not significantly predict changes in any of the three mediators (observed mindful emotion regulation, adaptive coping and maladaptive coping) at either timepoint, indicating that MYmind and medication produced similar changes in children’s emotion regulation. However, for sustained changes, adaptive coping showed a trending increase in the mindfulness condition relative to medication across all three child ADHD outcome models (Bs = 3.504–3.724, ps < 0.10). As for mediator–outcome associations, early increases in maladaptive coping (baseline to 2 months) predicted worse DBDRS inattention at 10 months (B = 0.033, *p* < 0.05). Similarly, sustained increases in maladaptive coping (baseline to 4 months) significantly predicted worse DBDRS inattention at 10 months (B = 0.043, *p* < 0.05) and showed a trending association with worse CBCL attention problems at 10 months (B = 0.202, *p* < 0.10). However, because treatment did not differentially affect maladaptive coping, these associations did not translate into significant indirect effects. None of the 24 specific indirect pathways tested in Model Set 1 were statistically significant. For all findings on child emotion regulation mediators, see Table 2.

#### 3.1.2. Model Set 2: Adolescent Self-Regulation

For adolescents ages 11 and older, we looked beyond the three mediators examined in all children and added adolescents’ self-reported mindfulness (CAMM) and healthy self-regulation (HSR) as mediators. Direct treatment effects showed a different pattern for adolescents compared to the full sample. At the 4-month follow-up, medication produced significantly greater reductions than mindfulness training for DBDRS hyperactivity/impulsivity (c′ = −1.212, *p* < 0.001) and CBCL attention problems (c′ = −3.330, *p* < 0.05), but not for DBDRS inattention (c′ = −0.717, ns). At the 10-month follow-up, the pattern shifted. CBCL attention problems showed the strongest direct effect favoring medication (c′ = −4.638, *p* < 0.01), while direct effects on DBDRS inattention (c′ = −0.279) and DBDRS hyperactivity/impulsivity (c′ = −0.706) were not significant (see Table 3).

Treatment significantly predicted increases in healthy self-regulation from baseline to 2 months, with adolescents in the mindfulness condition showing greater improvements than those receiving medication across all three outcome models (Bs = 0.420–0.459, ps < 0.05). Additionally, maladaptive coping showed a trending decrease in the mindfulness condition relative to medication for the DBDRS hyperactivity/impulsivity model (B = −2.898, *p* < 0.10).

Several mediator–outcome associations were found. Early increases in observed mindful emotion regulation predicted fewer DBDRS inattention symptoms at 4 months (B = 0.760, *p* < 0.05) and showed a trending association at 10 months (B = 0.646, *p* < 0.10). Early increases in adaptive coping showed a trending association with fewer DBDRS hyperactivity/impulsivity symptoms at 4 months (B = −0.045, *p* < 0.10). Contrary to our expectations, early increases in healthy self-regulation predicted more CBCL attention problems at 10 months (B = 3.739, *p* < 0.05), and sustained increases in healthy self-regulation similarly predicted more CBCL attention problems at 10 months (B = 2.660, *p* < 0.05). Sustained adolescent mindfulness (CAMM) showed a trending negative association with CBCL attention problems at 10 months (B = −4.216, *p* < 0.10). Additionally, sustained changes in maladaptive coping showed trending associations with both DBDRS inattention (B = 0.045, *p* < 0.10) and DBDRS hyperactivity/impulsivity (B = −0.038, *p* < 0.10) at 10 months, with opposite directions.

Despite significant individual a- and b-paths, their combination did not produce significant indirect effects for individual mediators. However, the total indirect effect for CBCL attention problems at the early timepoint showed a trending positive effect (1.434, *p* < 0.10), suggesting that the combined mediators may partially account for treatment differences, albeit in an unexpected direction. None of the 42 specific indirect pathways tested in Model Set 2 were statistically significant. For all findings on adolescent self-regulation mediators, see Table 3.

#### 3.1.3. Model Set 3: Parent-Level Mechanisms

We examined whether treatment effects on child ADHD symptoms operated through changes in parenting behaviors and parent psychological functioning. In the early models, where mediators were measured as change scores from baseline to 2 months, medication produced significantly greater reductions than mindfulness training at the 4-months follow-up for all three outcomes: DBDRS hyperactivity/impulsivity (c′ = −1.581, *p* < 0.001), DBDRS inattention (c′ = −1.451, *p* < 0.001) and CBCL attention problems (c′ = −5.033, *p* < 0.001). At the 10-month follow-up, between-group differences remained significant for DBDRS hyperactivity/impulsivity (c′ = −0.623, *p* < 0.05) and CBCL attention problems (c′ = −2.694, *p* < 0.05), while DBDRS inattention showed a trending effect (c′ = −0.513, *p* < 0.10). In the sustained models, where mediators were measured as change scores from baseline to 4 months, between-group differences at 10 months were significant for CBCL attention problems (c′ = −3.116, *p* < 0.05), while DBDRS measures showed trending effects (DBDRS H-I: c′ = −0.468, *p* < 0.10; DBDRS Att: c′ = −0.487, *p* < 0.10; see Table 4).

Treatment significantly affected multiple parent-level mediators. For early changes (baseline to 2 months), parents in the mindfulness condition showed significantly greater increases in self-compassion compared to those in the medication condition across all three outcome models (Bs = −0.374 to −0.378, ps < 0.01). Parents in the mindfulness condition showed a trend toward greater reductions in over-reactive parenting than those whose children were in the medication condition. (Bs = 0.162–0.176, ps < 0.10) Mindful parenting (IM-P) showed a trending increase in the mindfulness condition compared to the medication condition for two models (Bs = −0.065, ps < 0.10). For sustained changes (baseline to 4 months), the mindfulness condition showed significantly greater reductions in over-reactive parenting (Bs = 0.228–0.229, ps < 0.01) and significantly greater increases in mindful parenting (Bs = −0.113 to −0.114, ps < 0.05) compared to the medication condition.

Several mediator–outcome associations were observed in this model set. For early mediator changes (baseline to 2 months), increases in mindful parenting significantly predicted fewer child hyperactivity/impulsivity symptoms at 4 months (B = −1.611, *p* < 0.05) and fewer child CBCL attention problems at 4 months (B = −8.181, *p* < 0.05), with a trending association for CBCL attention problems at 10 months (B = −5.858, *p* < 0.10). Lax parenting showed a trending association with fewer DBDRS hyperactivity/impulsivity symptoms at 10 months (B = −0.444, *p* < 0.10). For sustained mediator changes (baseline to 4 months), increases in parental self-compassion significantly predicted fewer child DBDRS inattention symptoms at 10 months (B = −0.629, *p* < 0.05), and increases in mindful parenting showed a trending association with fewer child CBCL attention problems at 10 months (B = −4.651, *p* < 0.10). Changes in over-reactive parenting, general parental mindfulness (FFMQ) and lax parenting did not significantly predict child outcomes.

Despite significant treatment effects on mediators (a-paths) and multiple mediator–outcome associations (b-paths), none of the 45 specific indirect pathways tested in Model Set 3 produced statistically significant mediation effects. For all findings on parent-level mediators, see Table 4.

## 4. Discussion

### 4.1. General Discussion

In this paper, we examined potential mechanisms through which MYmind mindfulness training intervention affects child ADHD symptoms compared with methylphenidate. Prior reports established direct effects of MYmind on child ADHD symptoms [46,71,92]. Across 111 specific indirect pathways tested in 18 models, we found numerous significant individual pathways consistent with theoretical predictions, yet no complete mediation chains reached statistical significance.

Compared to methylphenidate, MYmind produced greater improvements in adolescent healthy self-regulation, parental self-compassion, mindful parenting and over-reactive parenting. The intervention’s theoretical model proposes that parallel parent and child mindfulness training should engage both child-level self-regulatory capacities and family-level parenting mechanisms [62,65,67], and our results are consistent with this proposition.

A reversed pattern emerged for maladaptive coping. Neither treatment differentially reduced it, but increases in maladaptive coping consistently predicted worse inattention in both early and sustained models. Maladaptive coping appears to be a robust correlate of attention difficulties that may require more focused intervention than either MYmind or methylphenidate currently provides.

Medication advantages were most pronounced at the 4-month follow-up, with significant effects favoring methylphenidate for all outcomes across all model sets. By 10 months, these direct effects attenuated. In the six-month interim between the 4- and 10-month follow-ups, families were in a naturalistic phase in which they were free to pursue any treatment they chose, including the treatment of the condition to which they were not originally assigned. In the full sample, only DBDRS hyperactivity/impulsivity maintained a significant direct effect favoring medication. In the adolescent-only models, CBCL attention problems showed a different pattern, with the medication advantage increasing from 4 to 10 months. However, the adolescent-only analyses included a smaller sample due to the addition of two mediators measured exclusively by adolescent self-report. These differential trajectories may therefore reflect differences in statistical power rather than meaningful developmental differences in treatment response. Children in the methylphenidate condition were in a structured treatment phase for the first 4 months, continuing to take medication and managed by regular check-ins with a psychiatrist. Children in the mindfulness condition were in an independent management setting between 2 and 4 months, practicing (or not) mindfulness without structured support other than a 16-week booster session.

These mediator–outcome associations are consistent with prior research. The importance of adolescent emotion regulation in ADHD is well established [21,24], and experimental evidence indicates that self-compassion reduces parental guilt and shame in response to challenging parenting events [93]. Our parent-level findings support theoretical models proposing mindful parenting and parental self-compassion as mechanisms of change in child mental health outcomes [62,67], with preliminary empirical support from an open trial of mindful parenting in clinical care [68]. Colonnesi et al.’s [94] findings offer longitudinal support for this, showing that parents’ capacity to attune to their child’s mental states predicted children’s social competence and behavioral outcomes.

Several hypothesized pathways were not supported. Treatment did not differentially affect children’s emotion regulation or coping strategies, nor adolescents’ dispositional mindfulness, when compared with methylphenidate. No treatment effects emerged for parents’ general mindfulness or lax parenting. Over-reactive and lax parenting, general parental mindfulness and adolescent dispositional mindfulness did not significantly predict ADHD outcomes; however, lax parenting did show a trending association with hyperactivity/impulsivity. None of the 111 specific indirect effects achieved statistical significance.

Despite the absence of significant specific indirect effects, we consider these results promising for mindfulness training as a viable approach for families of children with ADHD. Improvements in self-regulation and parenting are themselves clinically meaningful outcomes for family functioning regardless of their relationship to ADHD symptoms. Across all models tested, children and families in the MYmind condition improved as much as or more than those receiving medication on these constructs.

The observed pattern for parents’ self-compassion is noteworthy. MYmind produced significantly greater improvements in parental self-compassion from baseline to 2 months. In the sustained model, treatment no longer significantly predicted self-compassion, but self-compassion changes (baseline to 4 months) predicted fewer child DBDRS Inattention symptoms at 10 months. However, neither indirect pathway reached statistical significance. For mediation to be established, both the treatment-to-mediator path (a) and the mediator-to-outcome path (b) must be significant within the same model. In the early-change model, only the a-path (treatment to self-compassion) was significant. In the sustained-change model, only the b-path (self-compassion to ADHD outcomes) was significant. Whether this reflects insufficient power or a true absence of mediation cannot be determined from our data. Geurts et al. [41] reported a comparable pattern in that MBCT improved self-compassion in adults with ADHD, but the improvement did not mediate symptom reduction. Bögels et al. [95] found that parents rated self-care and self-nourishing attention among the areas most positively changed following mindful parenting training. Self-compassion may contribute to well-being and positive mental health without necessarily serving as a mechanism of symptom change.

Adolescents’ healthy self-regulation results are difficult to interpret. While MYmind produced significant improvements in adolescents’ healthy self-regulation compared to medication, greater HSR improvements were associated with more adolescent CBCL attention problems at 10-month follow-up, for both HSR early changes and sustained changes. Consistent with this, the total indirect effect for CBCL attention problems at the early timepoint showed a trending positive effect across all adolescent mediators combined, suggesting that the combined mediator pathways may have operated in an unexpected direction for this particular outcome measure. This counterintuitive association was specific to the CBCL and was not observed for DBDRS inattention or hyperactivity/impulsivity. The CBCL Attention Problems scale, unlike DSM-based measures, includes sluggish cognitive tempo items (e.g., daydreaming and appearing confused) that are empirically distinct from ADHD inattention symptoms [96]. The CBCL construct may therefore relate differently to healthy self-regulation than the criterion-based symptoms captured by the DBDRS. Given that 111 specific indirect pathways were tested, this isolated finding may represent a chance association or measurement-specific variance. Replication is needed before drawing conclusions.

### 4.2. Relation to the Prior Literature

Our findings add to the understanding gained from previous work on ADHD treatment mechanisms. The results partially overlap with Sibley et al.’s [39] review of psychosocial treatment mediators for ADHD. While they found that adolescent organization skills and parent–teen communication mediated outcomes, our results highlighted mindful parenting and self-compassion as promising parent-level mechanisms. Child-level self-regulatory capacities did not emerge as significant mediators in our study. The pattern of underpowered and null mediation findings is not unique to our work [35].

MBIs improved multiple outcomes without engaging measured mindfulness as a mediator, consistent with recent meta-analyses reporting that mindfulness-based interventions improve ADHD symptoms without necessarily improving self-reported mindfulness skills [42,43,44]. As Kosterman Zoller et al. [92] suggested, trait mindfulness may require longer-term practice to develop, or response-shift phenomena may obscure true changes. Alternatively, MBIs may work primarily through pathways other than mindfulness.

### 4.3. Strengths, Contributions and Limitations

Understanding the mechanisms through which psychosocial treatments for children with ADHD and their parents work can help improve treatment efficacy, inform personalization and clarify which intervention components are essential. This study provides the first direct comparison of mechanisms across pharmacological and psychosocial ADHD treatments, a priority identified by multiple reviews [35,38,39]. It tests whether mindfulness-based interventions work through their theoretically proposed mechanisms, a question raised by the finding that MBIs improve symptoms without necessarily improving measured mindfulness [42,43,44]. By examining mediators at multiple timepoints, it captures the temporal dynamics of treatment mechanisms, showing that some effects emerge early (e.g., parental self-compassion by 2 months), while others develop more gradually (e.g., mindful parenting and over-reactive parenting changes by 4 months).

The study included comprehensive measurement of potential mechanisms across child/adolescent and parent levels. The design incorporated both self-report and observational measures. Primary ADHD outcomes were based on parent observation of child behaviors, and observed mindful emotion regulation was assessed through independent observer coding of video-recorded parent–child interactions, reducing shared method variance. The study also drew on multiple informants (children’s self-report and reports from both parents), baseline assessments enabling change score analyses and multiple follow-up timepoints, allowing examination of both short-term and sustained mechanism engagement.

Several methodological limitations should be taken into consideration when interpreting these findings. Indirect effects are calculated by multiplying the a-path (treatment to mediator) and b-path (mediator to outcome) regression coefficients. Statistical uncertainty from both paths combines making indirect effects less precise than either path alone. This problem grows when multiple mediators are modeled simultaneously. The study was powered for its primary aim of detecting between-group differences in ADHD symptoms rather than for mediation specifically [97]. Most mediation studies in the literature are similarly underpowered [98]. Although our sample size exceeded thresholds for detecting medium indirect effects in single-mediator models, power analyses indicated that we had approximately 35–40% power to detect the small indirect effects actually observed, well below the conventional 80% threshold. Testing multiple mediators simultaneously further divided our statistical power and the partial clustering of parents within families reduced the effective sample size.

The adolescent subsample (*n* = 48) was particularly underpowered. And in models with five simultaneous mediators, the number of estimated parameters approached or exceeded the number of clusters, raising concerns about the reliability of between-level standard errors [91]. This was not the case for the full sample of parent-level models, where cluster-to-parameter ratios were more favorable. Taking these constraints into consideration, our null mediation findings should be interpreted as inconclusive rather than definitive evidence against mediation.

Our assessment schedule (baseline, 2, 4 and 10 months) may not have captured the temporal dynamics of mediational processes. Measurement intervals are often determined by convenience rather than theory [99], risking missed effects when intervals are misaligned with actual change processes [100,101].

Overlapping mediator and outcome measurement periods preclude definitive conclusions about temporal precedence. Reverse causation remains plausible, particularly for methylphenidate, for which pharmacological symptom control may precede improvements in coping and self-regulation. The lack of a waitlist or treatment-as-usual control group further constrains our conclusions [102]. We can conclude only that the examined variables did not explain differential treatment effects between MYmind and methylphenidate.

MYmind is a parent–child intervention that treats both participants at the same time, whereas methylphenidate, by contrast, treats only the child; it is an asymmetrical comparison in terms of number of family members receiving treatment. However, as methylphenidate lasted twice as long (16 weeks versus 8 weeks MYmind), it was also an asymmetrical comparison in the other direction in terms of duration of treatment. A symmetrical comparison of MYmind against Cognitive Behavior Training for child and parent was carried out by Wong et al. [103], showing similar effects for both treatments.

Combining the RCT and preference trial samples, while maximizing statistical power, introduces potential selection bias. Adherence to assigned treatments was monitored only for the first 4 months; subsequent treatment-seeking behavior may have influenced longer-term outcomes in ways for which we could not account.

The workbooks and written exercises of the MYmind program require a sufficient level of reading comprehension. To ensure children could engage with the materials, we set inclusion criterion for children with an IQ above 80. This comes with a trade-off in generalizability, as our results apply to a higher-functioning population and may not extend to children with ADHD and lower intellectual functioning.

Dual-parent participation in mindfulness training varied across families. Both parents were invited to attend the sessions, but participation was not randomly assigned, nor was it linked to parent gender. As a result, any differences in outcomes between single- and dual-parent participation, or between father or mother participation cannot be attributed to co-participation or parental gender alone, as these families may have differed in other meaningful ways. Whether parental co-participation or father versus mother involvement shapes the strength of family-level treatment effects is a question worth examining in future work.

The absence of changes in Child and Adolescent Mindfulness Measure for adolescents’ self-reports within the mindfulness condition may be due to a measurement issue rather than an actual absence of change. The CAMM has been shown to be a cross-cultural, psychometrically validated trait-based measure [83,84,104] but can become problematic when measured longitudinally with children that have participated in mindfulness training. The MYmind classes and exercises can increase children’s awareness and comprehension of mindfulness traits. De Bruin et al. [104] describe this as a process of four stages of learning in which children progress from initial unconscious incompetence to conscious competence, where they become aware of their unmindful traits and, as a result, might rate themselves lower than before training. The response-shift effect [105,106] could lead to stable or even decreased self-reporting when actual mindfulness traits have improved due to a child’s understanding of the question changing rather than accurate recording of traits across time. Bartos et al. [107] found that when bias of mindfulness self-reports after training is statistically corrected for, actual intervention effects are found to be twice or more than uncorrected scores indicate. It is notable that in our adolescent subsample, video-observed improvements in mindful emotion regulation predicted reductions in ADHD symptoms, while CAMM self-reports did not. This lends credence to the idea that behavioral observation may better capture trait changes than self-reports.

This contrast points to a broader measurement challenge. Trait-based mindfulness items may be particularly vulnerable to recalibration after training as accurately measuring one’s own mindfulness requires observational abilities and awareness that training develops [108]. A systematic review of instruments that measure mindfulness found that none had examined this potential response-shift in respondents’ understanding of items following meditation training [109]. Focusing on concrete behavioral items rather than abstract trait questions may reduce susceptibility to recalibration. An additional approach could be ecological momentary assessment, in which participants respond to brief questions about their experience throughout daily life, capturing mindfulness as it occurs rather than through retrospective global judgment [110].

All parental self-report measures of mindfulness have the same recalibration issues as children’s measures. Parents who developed greater mindfulness skills by having followed the MYmind training are likely to have under reported their own mindfulness at follow-ups relative to baseline. While we observed improvements on all three parental scales of mindfulness within the parent group, the actual growth may have been greater than what was reported.

Testing 111 specific indirect pathways increases the possibility that any significant paths emerged by chance, while the stringent requirement that both a-paths and b-paths reach significance may have been overly conservative given our sample size. Future research should incorporate larger samples, intensive longitudinal assessment during the intervention period and a no-treatment control condition to enable stronger causal inferences about mechanisms of change.

## 5. Conclusions

This is among the first studies to examine potential mechanisms for effects of a mindfulness training intervention on child ADHD symptoms. The absence of significant indirect effects means results should be interpreted with caution. Nevertheless, MYmind engaged multiple theoretically relevant mechanisms that separately predicted outcomes. This pattern of findings strengthens the case for continued research into mindfulness-based interventions for families of children with ADHD.

The findings also highlight the persistent methodological challenges facing ADHD mechanism research. Although we tested 111 specific indirect pathways at multiple timepoints, the complexity of the multi-step causal chains may have contributed to statistical power remaining insufficient to detect what may have been small-to-medium indirect effects. Future research may benefit from larger samples; more sensitive measurement approaches; or alternative analytic strategies, such as component-wise evaluation of intervention effects.

The evidence base for family-based mindfulness interventions for ADHD is still developing, but the present findings suggest these approaches engage meaningful processes at both the parent and child level. For families of children with ADHD seeking alternatives to methylphenidate as medication, mindfulness-based family interventions such as MYmind offer a promising approach that engages both parent and child in developing skills for attention, emotion regulation and responsive parenting. The precise mechanisms through which these benefits emerge remain to be fully understood.

## Data Availability

Data from this study are stored in the University of Amsterdam repository.

## Supplementary Materials

The following supporting information can be downloaded at https://www.mdpi.com/article/10.3390/children13030434/s1, Table S1: TREND Statement Checklist; Table S2: Descriptive statistics for child-related mediators and outcomes across models; Table S3: Descriptive statistics for parent-related mediators and outcomes across models.
