# Supplementary material for: Mechanisms of Change in Mindfulness-Based Family Intervention (MYmind) Versus Methylphenidate for Childhood ADHD: A Randomized Controlled Trial
Source: Children (Basel). 2026 Mar 23;13(3):434. doi: 10.3390/children13030434 (PMC13025426; doi:10.3390/children13030434)
Supplement: Supplementary file 1 [file children-13-00434-s001.zip › children-4178055-supplementary.pdf]

Supplementary materials

Table S1. TREND Statement Checklist.

Title and Abstract	Section
Information on how units were allocated to interventions	Title; Abstract
Structured abstract recommended	Abstract
Information on target population or study sample	Abstract
Introduction	
Scientific background and explanation of rationale	1.1–1.4
Theories used in designing behavioral interventions	1.6–1.8
Methods	
Eligibility criteria for participants	2.1
Method of recruitment	2.1
Recruitment setting	2.1
Settings and locations where data were collected	2.2
Content: what was given?	2.3.1; 2.3.2
Delivery method	2.3.1; 2.3.2
Unit of delivery: how were subjects grouped?	2.3.1
Deliverer: who delivered the intervention?	2.3.1; 2.3.2
Setting: where was the intervention delivered?	2.3
Exposure quantity and duration	2.3.1; 2.3.2
Time span for delivery	2.3.1; 2.3.2
Activities to increase compliance/adherence	2.3.1; 2.3.2
Specific objectives and hypotheses	1.9
Clearly defined primary and secondary outcome measures	2.4.1–2.4.4
Methods used to collect data and quality enhancement	2.4
Information on validated instruments	2.4.1–2.4.4
How sample size was determined; interim analyses	2.1; 4.3
Unit of assignment	2.1–2.2
Method used to assign units to conditions	2.2
Aspects to minimize bias due to non-randomization	2.1; 2.2
Blinding of participants, administrators, assessors	N/A
Description of smallest unit analyzed	2.5
Method to account for unit of analysis ≠ unit of assignment	2.5
Statistical methods for primary comparisons	2.5

Methods for additional analyses	2.5
Methods for imputing missing data	2.5
Statistical Software	2.5
Results	
Flow of participants through each stage	2.1; 2.2; Figure 1
Description of protocol deviations	2.3.1; 2.3.2
Dates defining periods of recruitment and follow-up	2.2
Baseline demographic and clinical characteristics	2.1; Tables S2 and S3
Baseline comparisons: lost to follow-up vs. retained	N/A
Comparison between study population and target population	N/A
Data on study group equivalence at baseline	N/A
Number of participants in each analysis	Tables 2–4
Intention-to-treat or non-complier handling	2.1; 2.2
Summary of results with effect sizes and CIs	3.1.1–3.1.3; Tables 2–4
Null and negative findings included	3 (all subsections)
Results from testing pre-specified causal pathways	3 (all subsections)
Summary of other analyses (subgroup, exploratory)	3.1.1–3.1.3
Summary of adverse events or unintended effects	2.3.2
Discussion	
Interpretation considering hypotheses, bias, limitations	4.1; 4.3
Discussion of causal pathways/alternative mechanisms	4.1
Success/barriers to implementation, fidelity	2.3.1
Research, programmatic, or policy implications	4.3; 5
Generalizability/external validity	4.3
General interpretation in context of current evidence	4.2; 5

Table S2. Descriptive Statistics for Child-Related Mediators and Outcomes Across Models.

**Sample**	**Variable**	**M**	**SD**	**N**
All Children	Mediators: Early Changes (BASELINE→2 MONTHS)			
	Mindful ER Discussion Task change	0.060	0.571	141
	Adaptive Coping change	1.000	9.130	192
	Maladaptive Coping change	−0.960	7.268	192
All Children	Mediators: Sustained Changes (BASELINE→4 MONTHS)			
	Adaptive Coping change	1.730	10.426	188

	Maladaptive Coping change	−0.870	8.039	188
All Children	Outcomes: DBDRS Attention			
	DBDRS Attention change BASELINE→4 MONTHS	−1.300	1.856	198
	DBDRS Attention change BASELINE→10 MONTHS	−1.640	1.648	178
All Children	Outcomes: DBDRS Hyperactivity-Impulsivity			
	DBDRS Hyperactivity-Impulsivity change BASELINE→4 MONTHS	−1.120	1.638	198
	DBDRS Hyperactivity-Impulsivity change BASELINE→10 MONTHS	−1.370	1.513	178
All Children	Outcomes: CBCL Attention			
	CBCL Attention change BASELINE→4 MONTHS	−4.720	7.620	198
	CBCL Attention change BASELINE→10 MONTHS	−5.650	7.927	175
Subsample Adolescents	Mediators: Early Changes (BASELINE→2 MONTHS)			
	Mindful ER Discussion Task change	0.020	0.601	66
	Adaptive Coping change	1.610	9.542	80
	Maladaptive Coping change	−0.110	5.857	80
	Mindfulness (CAMM) change	0.090	0.569	83
	Healthy Self-Regulation change	0.350	0.687	83
Subsample Adolescents	Mediators: Sustained Changes (BASELINE→4 MONTHS)			
	Adaptive Coping change	2.230	10.643	75
	Maladaptive Coping change	−1.430	9.085	75
	Mindfulness (CAMM) change	0.240	0.627	74
	Healthy Self-Regulation change	0.470	0.579	74
	Adaptive Coping change	2.230	10.643	75

Subsample Adolescents	Outcomes: DBDRS Attention			
	DBDRS Attention change BASELINE→4 MONTHS	−1.470	1.922	83
	DBDRS Attention change BASELINE→10 MONTHS	−1.510	1.536	74
Subsample Adolescents	Outcomes: DBDRS Hyperactivity-Impulsivity			
	DBDRS Hyperactivity-Impulsivity change BASELINE→4 MONTHS	−1.300	1.611	83
	DBDRS Hyperactivity-Impulsivity change BASELINE→10 MONTHS	−1.420	1.452	74
Subsample Adolescents	Outcomes: CBCL Attention			
	CBCL Attention change BASELINE→4 MONTHS	−4.820	6.399	82
	CBCL Attention change BASELINE→10 MONTHS	−4.010	6.688	73

Table S3. Descriptive Statistics for Parent-Related Mediators and Outcomes Across Models.

Sample	Variable	M	SD	N
Parent	Mediators: Early Changes (BASELINE→2 MONTHS)			
	Over-reactive Parenting (PS) change	−0.180	0.592	129
	Lax Parenting (PS) change	−0.010	0.526	129
	Mindfulness (FFMQ) change	0.060	0.276	129
	Mindful Parenting (IM-P) change	0.060	0.224	129
	Self-Compassion (SCS) change	0.130	0.685	128
Parent	Mediators: Sustained Changes (BASELINE→4 MONTHS)			
	Over-reactive Parenting (PS) change	−0.260	0.522	129
	Lax Parenting (PS) change	−0.090	0.538	129
	Mindfulness (FFMQ) change	0.090	0.279	129

	Mindful Parenting (IM-P) change	0.090	0.265	129
	Self-Compassion (SCS) change	0.180	0.686	128
Parent	Outcomes: DBDRS Hyperactivity-Impulsivity			
	DBDRS Hyperactivity-Impulsivity change BASELINE→4 MONTHS	−1.100	1.669	138
	DBDRS Hyperactivity-Impulsivity change BASELINE→10 MONTHS	−1.310	1.422	125
Parent	Outcomes: DBDRS Attention			
	DBDRS Attention change BASELINE→4 MONTHS	−1.300	1.863	138
	DBDRS Attention change BASELINE→10 MONTHS	−1.600	1.585	125
Parent	Outcomes: CBCL Attention			
	CBCL Attention change BASELINE→4 MONTHS	−4.930	7.724	137
	CBCL Attention change BASELINE→10 MONTHS	−5.760	7.198	122
